# Implementation of the first Objective Structured Practical Examination in veterinary medicine in Spain: a two-year experimental study

**DOI:** 10.3389/fvets.2025.1618069

**Published:** 2025-08-04

**Authors:** Lara Carrasco, Gema González, Maria Utrilla, Laura Rico-San Román, Antonio Magro, Natividad Pérez-Villalobos, Bárbara Martín-Maldonado

**Affiliations:** Department of Veterinary Medicine, School of Biomedical and Health Sciences, Universidad Europea de Madrid, Villaviciosa de Odón, Spain

**Keywords:** skills assessment, practical evaluation, students' perception, educational improvements, exam methodology, veterinary degree

## Abstract

**Background:**

In Veterinary Medicine, traditional methods for evaluating technical skills, such as multiple-choice exams or short questions, have limitations in assessing practical competencies. The Objective Structured Clinical Practical Examination (OSPE) has been successfully implemented in other Health Sciences degrees to improve objectivity and minimize bias in skill evaluation. This study aimed to assess the outcomes of implementing OSPE for evaluating Veterinary students' technical skills and to analyze students' perceptions and satisfaction with the method.

**Methods:**

Over two academic years, OSPE was introduced as an evaluation tool for Veterinary students, featuring four stations: Clinical Examination, Blood Sample Extraction, Anesthesia, and Surgery. Student performance was recorded, and their perceptions were gathered through voluntary surveys. Descriptive statistics were applied to analyze the exam results and survey responses.

**Results:**

A total of 144 students participated, with 93.8% passing the OSPE on their first attempt. Among the stations, the Surgery station received the highest scores both in student performance and survey feedback. Overall, 96% of students considered OSPE a positive evaluation method, and 92.7% acknowledged its effectiveness in assessing practical skills.

**Conclusions:**

The OSPE has significantly improved the evaluation of practical skills in Veterinary Medicine, offering an objective and structured approach that enhances learning strategies. Students demonstrated high satisfaction with the method, which also resulted in favorable exam outcomes.

## 1 Introduction

The assessment of students during their degree studies frequently relies on written tests to evaluate cognitive ability and practical performance tests to assess knowledge and competence. However, these methods often fail to evaluate practical skills adequately (1). Developing practical skills is a crucial aspect of the veterinary profession, yet the literature on implementing objective assessments for evaluating these skills is very limited.

The complexity and continuity of teaching-learning methods in university education require reliable assessment mechanisms (2). Traditional practical examinations have been subjective, primarily assessing cognitive ability while neglecting actual skills and communication abilities in settings (3–5). Effective assessments in medical education must comprehensively address knowledge, skill, and attitude. However, traditional methods fall short in evaluating psychomotor and communication skills and are prone to examiner variability, thus reducing reliability (6). Uniform and reliable assessment methods are crucial for achieving learning objectives, as learner performance indirectly measures teaching effectiveness. The Objective Structured Practical Examination (OSPE) addresses these issues by structuring and assessing practical skills to minimize subjectivity and allows for directly demonstrating applied knowledge and skills rather than solely assessing knowledge (7, 8). Consequently, this test method provides evidence of what students can do rather than what they know.

The OSPE derives from the Objective Structured Clinical Examination (OSCE), introduced in 1975 by Harden and his colleagues at the University of Dundee (Scotland) to enhance objectivity in clinical examinations. OSCEs are widely used to assess clinical skills, and they typically evaluate critical thinking and the student's ability to interpret clinical parameters (9). In 1979, after introducing some modifications, the first OSPE was reported, focusing solely on assessing the mechanical performance of clinical or pre-clinical procedures, without evaluating diagnostic reasoning or interpretation (10). OSPE assessment involves a series of stations where students, using standardized (or simulated) patients or instruments, perform specific tasks such as physical examination, equipment usage in experiments, sequence of steps followed in experiments, clinical skills, etc. (1, 11). In each station, candidates are given a specific task, carefully structured to include elements from the entire curriculum and a wide range of skills (12). All candidates are assessed using the same stations and receive a score for each step they perform correctly. The evaluation should be done according to a list of positive and negative indicators developed when designing the stations. This list, or rubric, ensures skill competency and reduces examiner bias, making the assessment more objective (3, 4). This approach aligns assessment methods with educational objectives and enables a comprehensive evaluation of pedagogical goal attainment (6).

Currently, the OSPE is predominantly used in Health Sciences Schools, often in conjunction with traditional methods. The OSPE's effectiveness in evaluating practical skills highlights its superiority over traditional methods, aligning with modern medical education requirements (2). In this context, it is anticipated to eventually replace these subjective assessment methods (8). The use of OSPE in disciplines such as Medicine, Nursing, or Dentistry has shown significant advantages, such as covering a wide range of knowledge and skills with minimal variability (5). Recent research shows that the assessment method affects student learning, with OSPE leading to better performance than conventional methods, demonstrating its relevance and feasibility for undergraduate training (6).

Despite its advantages, the OSPE requires significant planning, coordination, and labor, often necessitating numerous assessors (13). However, various studies demonstrate a significant difference in scores obtained via OSPE compared to conventional practical examinations, indicating its superior effectiveness and validity by eliminating inter-examiner variation and bias (2, 5, 13).

Given the critical importance of practical skills in Veterinary Medicine, an OSPE was implemented in the “Introduction to Veterinary Clinics” (IVC) from the second year of the Veterinary Degree at the Universidad Europea de Madrid. This study aimed to report the design, implementation, and assessment of the results of the first two promotions (2022–2023 and 2023–2024).

## 2 Methodology

### 2.1 Participants

The OSPE was given to all second-year Veterinary Degree students who have enrolled in the subject Introduction to Veterinary Clinic (IVC) for 2022–2023 and 2023–2024. In both years, the OSPE took place at the end of the second semester after finishing the theoretical and practical content of the course. All the participants were informed at the beginning of the subject that the OSPE would be considered a part of their mandatory evaluation process.

### 2.2 OSPE: content and format

The content evaluated in the OSPE consisted of practical seminars at three locations: the Simulated Veterinary Hospital of the Universidad Europea de Madrid, one of the first in Europe; a pre-professional internship at an animal shelter; and an internship at a farm school. The practical workshops in the simulated hospital amounted to 20 h per student, divided into 16 sessions. The pre-professional practices in an animal shelter with dogs and cats consisted of 20 h per student. In addition, there was an internship at a farm school handling cows, horses, and other domestic animals for more than 5 h per student.

A team of five Veterinary teachers developed an OSPE consisting of simulated technical actions, the most authentic to daily clinical practice, divided into four stations or simulated scenarios. At the end of the semester, the OSPE took place at the simulated hospital. The students waited together in a room until they were called to perform the OSPE. Subsequently, they started in small groups and rotated through the four scenarios in a systematic and orderly manner during the OSPE. The stations were physically separated so that the students could not see the others, and a teacher was always carefully observing each student's actions. Each station had several different scripts of the same difficulty level, and students had to silently perform the one randomly assigned to them within a maximum of 5 min.

The first station, “Clinical Examination”, consisted of three possibilities: a horse, a dog, and a cow simulator. In this station, different parameters or tests key to the clinical examination of these animals were asked to be performed on the participants, such as the point of auscultation of the mitral valve in the dog, and of the ilio-cecal valve in the horse.

The second station “Blood Sample Extraction” also had several possibilities: dog's cephalic, saphenous, and jugular veins, and horse's jugular vein simulators. In addition, the script also requested the selection of different sample preservation tubes according to the required analyses.

The third station, “Anesthesia”, tested different essential anesthesia skills: the ability to intubate a dog, the detection of peripheral pulse, the assembly of an anesthetic circuit, and the recognition of different structures of an anesthesia machine are some of them. This station consisted of two substations: one of them with a dog manikin where different peripheral veins could be appreciated and a dog head simulator for intubation, and the other one with an anesthesia machine and monitor with preset values like those of an anesthetized dog or cat. Students are evaluated in both substations.

The last station “Surgery” consisted of a sterile gown, sterile gloves, surgical material, a skin pad for suture training, and sutures. First, they had to demonstrate their skills in sterile dressing with gowns and gloves (open or closed technique as the script indicated). Then, they had to perform a suture, choosing and using the appropriate surgical material. Likewise, they had to maintain sterility during the whole process.

### 2.3 Scoring

Each OSPE scenario was scored according to the total, partial, or null performance of the different actions detailed in the rubric of each of the scripts. If the performance of the action was correct, the score was one, if it was partially correct, the score was 0.5, and if it was incorrect, it was 0. Each of the rubrics consisted of different indicators: the rubric for the first station had eight indicators, and those for the second, third, and fourth stations had 10 sections. Each indicator described a concise and detailed action to make the evaluation as objective as possible. Each teacher evaluated one student at a time and completed the rubric as he or she carried out the actions described in the script for each scenario.

All sections of the rubric were weighed equally. Once the scenario was completed, the score for each of the items was summed and scored out of 10. The final OSPE score consisted of the average of the rubric scores for each station. To pass the OSPE, the final grade had to be higher than 5 out of 10.

### 2.4 Surveys

Following the completion of the OSPE, a free and voluntary survey was distributed to all participants to gather their perceptions regarding its organization and execution, as well as the organization of the course and practical workshops (Online Resource 1). The survey included closed-ended questions about the organization of workstations, the time limits for completing tasks, the alignment of the tasks assessed in the OSPE with those performed throughout the semester, the simulators' usefulness, and their realism compared to actual clinical practice. It also addressed the duration and usefulness of pre-professional practices during the semester. Moreover, the students were asked to answer, with a maximum of three options per question, about the emotions and perceptions experienced during the whole OSPE process: on one hand, the feelings in the moments before and during the OSPE, and on the other hand, the feelings after the OSPE. All surveys were submitted anonymously, and the responses were entered into a database for subsequent analysis.

### 2.5 Data analysis

The results obtained from the OSPE were analyzed collectively to derive the basic statistical parameters (mean, median, mode, standard deviation, standard error, and 95% confidence intervals). Additionally, the survey responses were entered into a database for analysis. The Excel^®^ software package (Microsoft 365, version 2406) was used for all the analyses and the graphics creation.

## 3 Results

A total of 144 students were evaluated through the OSPE methodology: 61 in 2023 and 83 in 2024. However, as the survey was voluntary, only 102 (70.8%) students completed it: 29 from the 2023 class (47.5%) and 73 from the 2024 class (87.9%).

In general, the evaluation system that the OSPE represented was considered positive for 96% of the students, and 92.7% of them highlighted its usefulness in assessing their practical skills. Moreover, all the practical sessions done during the semester, and the performance of the OSPE, allowed all the students (100%) to become aware of the practical skills necessary for the development of the veterinary profession.

**Figure 1:**
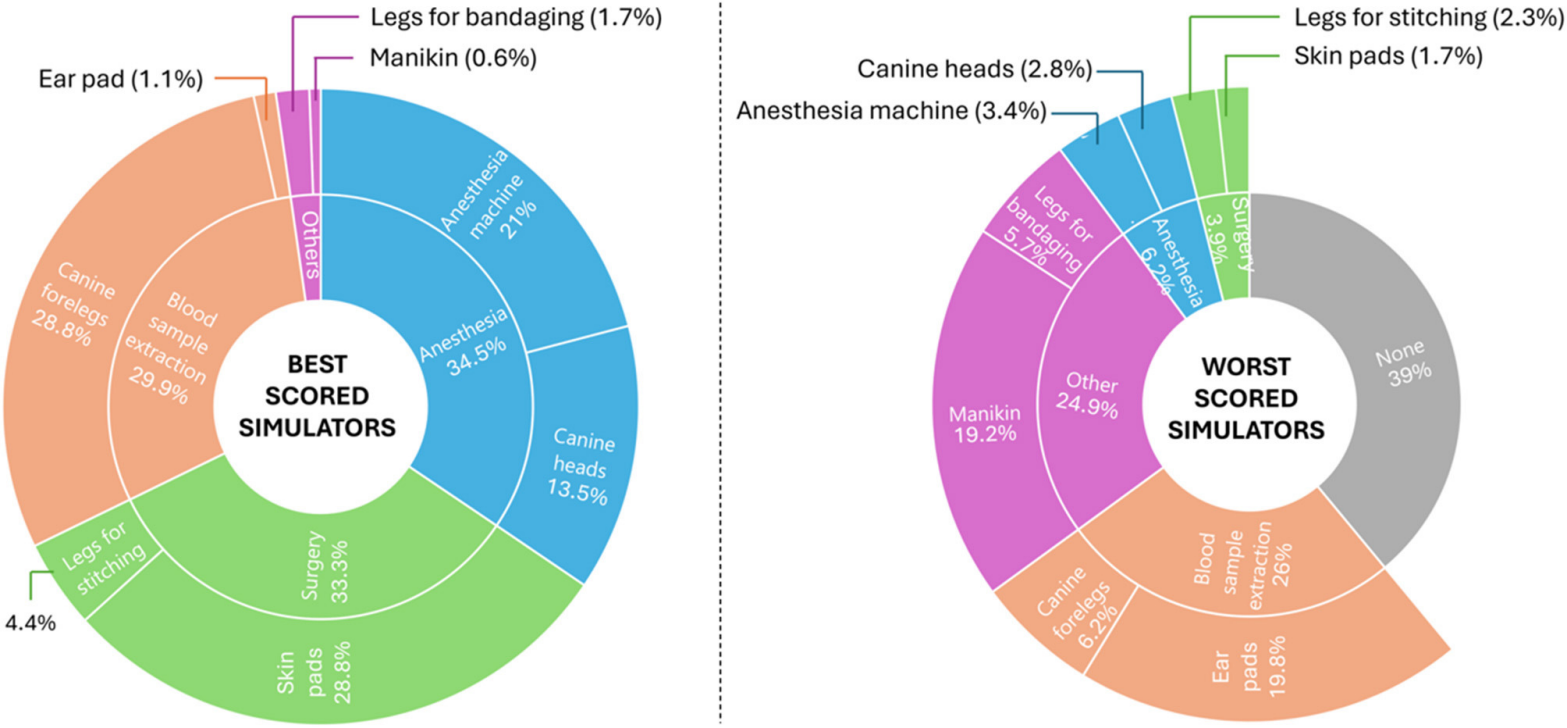
Assessment of the more useful and useless simulators (best and worst scored simulators, respectively) employed during the task-training workshops.

During the semester, students attended different task-training sessions to develop their automatic skills in some procedures (e.g. extracting a blood sample, giving stitches, or airway intubating) with specific veterinarian simulators. The employment of these simulators was helpful for 92% of the students in acquiring their technical abilities, and 89.1% of them considered them sufficiently realistic for the function they had. The practical sessions with simulators have favored the development of the skills and knowledge necessary for the resolution of the OSPE for 95% of the participants. In these workshops, the simulators better rated were canine forelegs for blood extraction (28.8%) and stitches skin pads (28.8%), followed by the anesthesia machine (20.9%) and canine heads for air intubation (13.6%). Despite 39% of the students considered all the simulators useful, others classified some of them as useless, such as the ear pads for blood extraction (19.8%) and the canine manikin (19.2%) (Figure 1).

**Figure 2:**
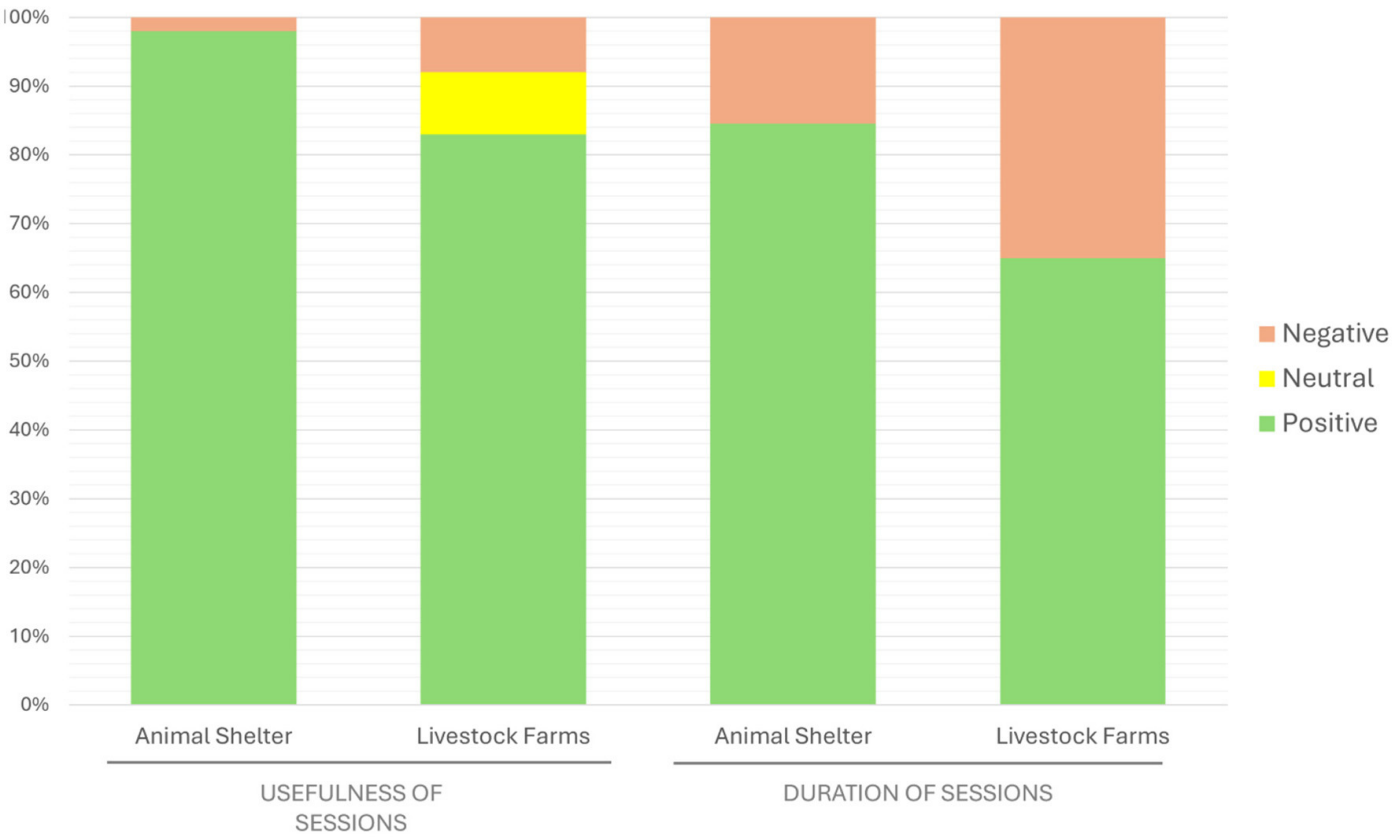
Assessment of the usefulness and duration of animal shelter and livestock farm pre-professional sessions during the semester. “Usefulness” or “good duration” of sessions were considered positive; “neither agree nor disagree” was considered neutral; and “useless” or “scarce” were considered negative answers.

Once they had practiced all these techniques, they attended pre-professional sessions at a companion animal shelter and different livestock farms. Most students reported that sessions at the animal shelter were useful, and their duration was suitable for strengthening their skills. In contrast, 35% of the participants opined that the duration of sessions in the livestock farms was insufficient (Figure 2).

**Figure 3:**
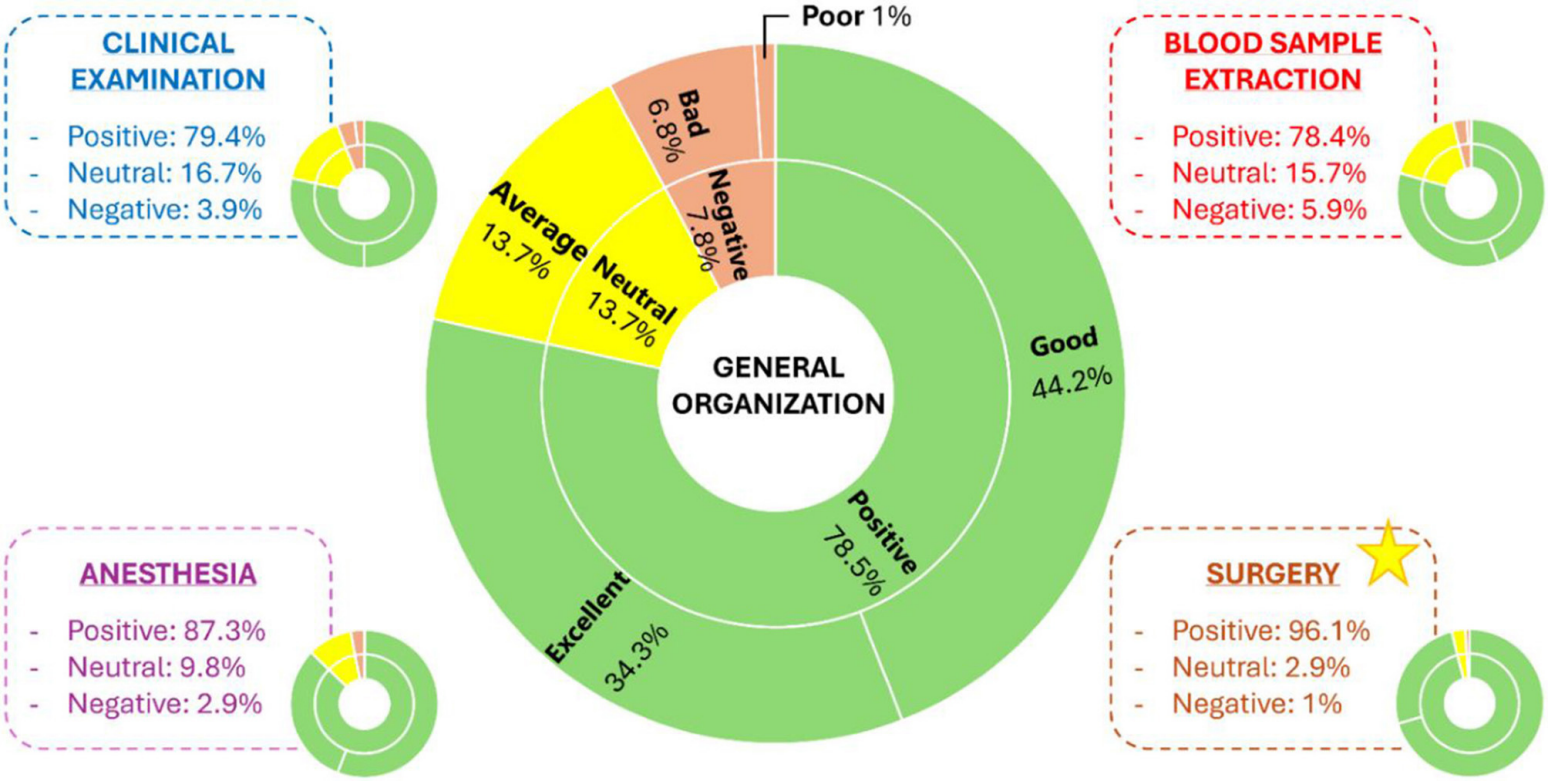
General and specific organization scores of the OSPE and each station, respectively. The yellow star marks the station with the best score. For all the graphics: in green, “Positive” answers included “Excellent” and “Good” organization; in yellow, “Neutral” answers meant “Average” organization; and in orange, “Negative” answers included “Bad” and “Poor” organization.

Organization of the final OSPE was perceived as good by 78.4% of the participants, with slight differences between the four stations. When specifically asked about the organization of each OSPE station, the surgery station received the highest score compared to the others, which received very similar scores (Figure 3). While some students reported scarce time to perform all the tasks from each workstation (35%), the whole OSPE duration was defined as good by 89% of the participants. Moreover, 92% of the students said that workstations were sufficiently realistic. However, of the remaining students, 75% considered that “Blood sample extraction” was the less realistic of all.

**Figure 4:**
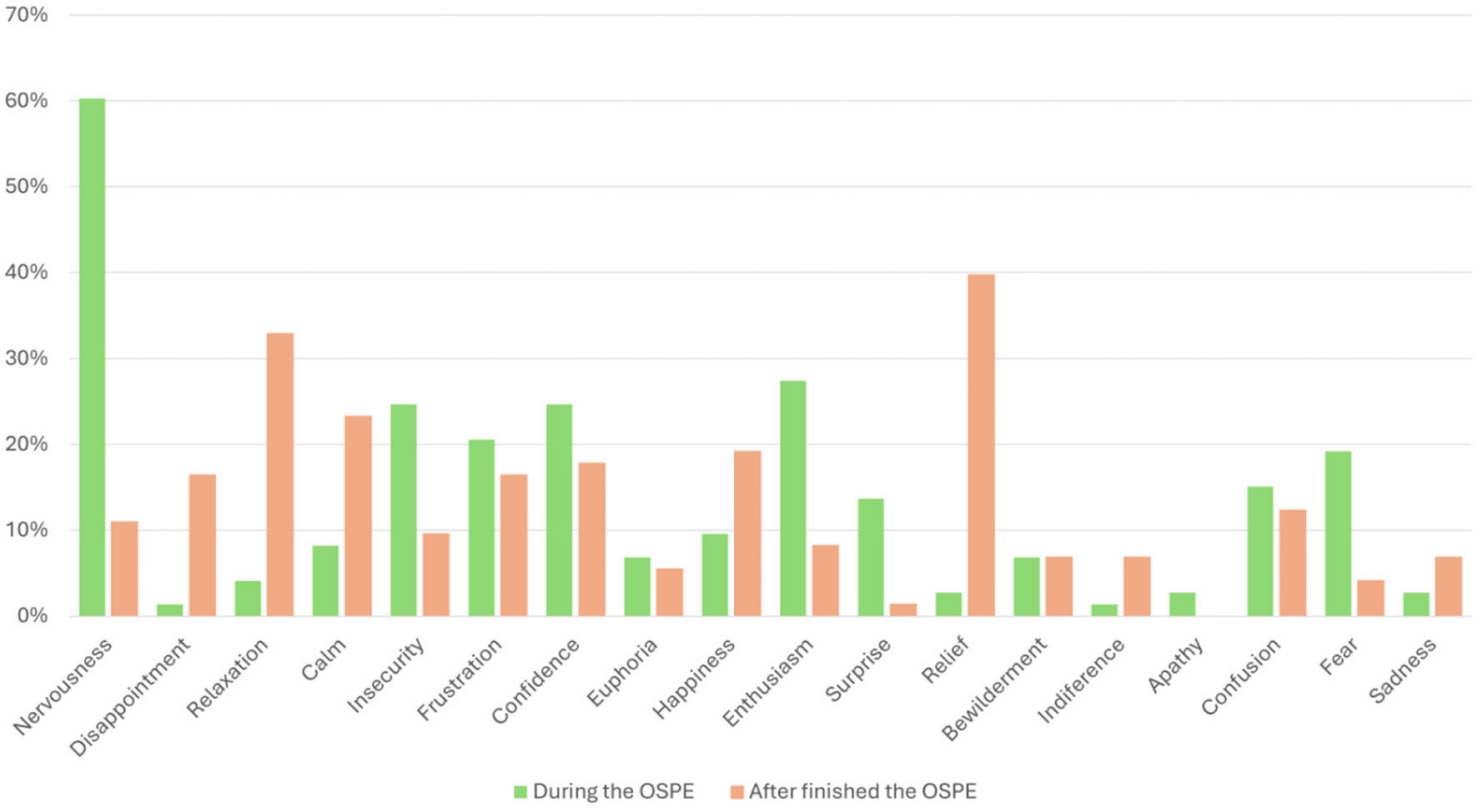
Distribution of emotions reported by students during the OSPE and after the OSPE.

The results of the emotions are detailed in Figure 4. It should be mentioned that in the moments immediately before and during the OSPE, 60.3% of the students stated they were nervous. Regarding the other emotions, 20–30% of the students felt insecurity (24.7%), frustration (20.6%), enthusiasm (27.4%) or confidence (24.7%), and between 10 and 20% of the students felt surprise (13.7%), confusion (15.1%) or fear (19.2%). However, at the end of the OSPE, more mixed results were observed among the students, with the most prominent emotions being relief (39.7%) and relaxation (32.9%). Of the other emotions, the results indicated that 15–23% of the students felt disappointed (16.4%), calm (23.3%), frustrated (16.4%), confident (17.8%), and happy (19.2%) after completing the test.

**Figure 5:**
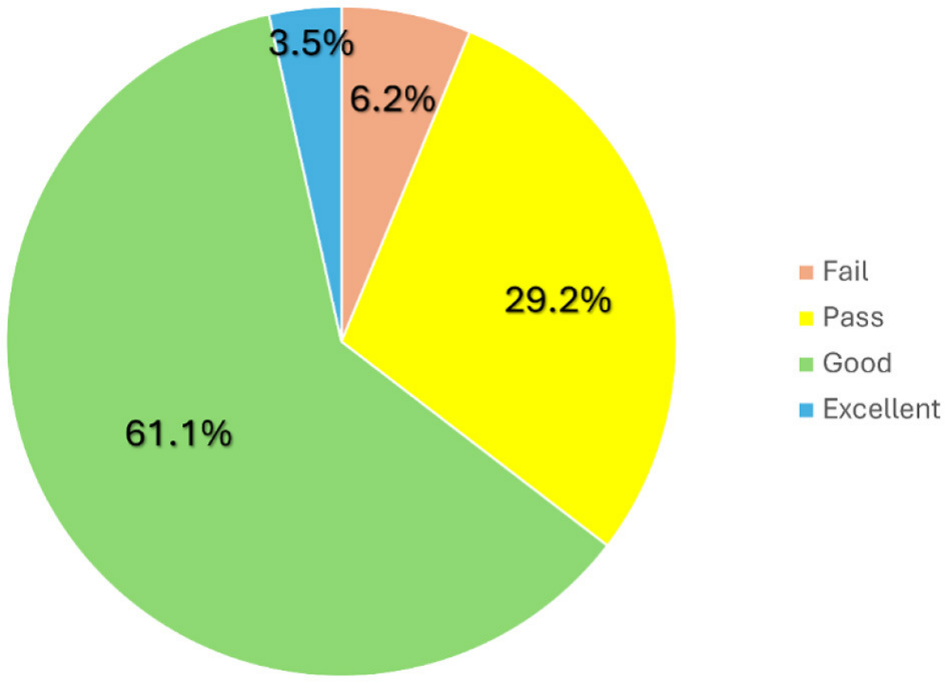
Distribution of the grades obtained by the 144 students who took the OSPE on the first attempt, where “Fail” includes scores from 0/10 to 4.9/10, “Pass” from 5/10 to 6.9/10, “Good” from 7/10 to 8.9/10, and “Excellent” from 9/10 to 10/10.

Finally, regarding the OSPE results, 135 of the 144 students (93.8%) passed the exam on their first attempt (Figure 5). Among all the students, the minimum score was 3/10, the maximum 9.5/10, the mean was 7.1/10, and the mode was 8/10. When analyzing the specific results from each station, the station with the best mean was “Surgery”, while the best mode was observed in the station “Anesthesia”. In contrast, “Clinical Examination” had the poorest results (Table 1).

**Table 1:** Specific results obtained by the 144 students in each station at their firstattempt.

**Parameters**	**Station 1: clinical Examination**	**Station 2: blood sample extraction**	**Station 3: anesthesia**	**Station 4: surgery**	**Global results**
Mean (CI_95%_^*^)	5.7/10 (5.4–6.1)	6.6/10 (6.4–6.9)	8/10 (7.7–8.3)	8.1/10 (7.9–8.4)	7.1/10 (6.9–7.3)
Median	6/10	6.8/10	9/10	8/10	7.2/10
Mode	6/10	7.5/10	10/10	9/10	8/10
Minimum score	1/10	2/10	1.5/10	3/10	3/10
Maximum score	10/10	10/10	10/10	10/10	9.5/10

^*^CI_95%_: 95% confidence interval.

## 4 Discussion

Since 1956, educational goals have been clustered into six major categories defined by Benjamin Bloom: Knowledge, Comprehension, Application, Analysis, Synthesis, and Evaluation. In healthcare degrees, practical assessments must evaluate not only Knowledge but all the major categories in Bloom's taxonomy (14). The traditional practical evaluations covered only the Knowledge and Comprehension domains with multiple choice questions (MCQ) tests, essays, or reports. In some cases, direct observation of the student performing a specific procedure was implemented, but as they were non-structured assessments with rubrics, they could not be objective and unbiased. The OSPE offers an unbiased and objective evaluation of the applicant's skills in standardized or simulated conditions (15). However, OSPE-based assessments have not been implemented in Veterinary Medicine. Thus, to our knowledge, this is the first OSPE in Veterinary Medicine reported in the indexed scientific literature, although unpublished or non-indexed reports may exist.

Before performing the OSPE, students must learn to use and practice with the instruments on which they will be evaluated. Simulators were very positively rated by students except for two of them: manikins and ear pads. For the first one, the simplicity of the simulator could be a determinant of the poor rate given by the students. For the second one, blood sampling from ear veins is difficult due to their small gauge: they are narrow, thin, and easy to collapse and rupture (16, 17). So, this situation could lead the students to frustration. Overall, the practical sessions with simulators allowed students to learn in a safe and controlled environment, without causing harm to animals, and effectively prepared them for their subsequent pre-professional training (18).

Among all the OSPE stations, “Blood sample extraction” was considered the least realistic, probably due to the logistics of the station and the simulators employed, since there is a big difference between taking a blood sample from a foreleg simulator and from a real dog that is nervous, frightened, or in movement. In contrast, “Anesthesia” and “Surgery” were the most realistic stations, which agrees with the degree of development and detail of the surgical simulators available in veterinary medicine. Furthermore, the use of these simulators during the first phases of learning is of great importance to ensure the animal welfare of the students' future patients.

Through our survey, we realized that the majority of our students regarded the OSPE as a positive experience, which agrees with previous studies (19, 20). However, Barreto-Mejía et al. (21) reported that students from Colombia preferred traditional didactic strategies instead of constructivism-based learning methods, probably due to the stress and anxiety of a new constructive evaluation tool. But, while some authors reported these negative emotions in their students, others considered the OSPE a less stressful methodology than traditional ones (5, 19, 22). It is important to mention the rise of stress-related suicide among university students, so the search for less stressful evaluation methods like the OSPE becomes a priority (5). Moreover, there is a confirmed correlation between anxiety and poor performance at an OSPE or OSCE (22). In this context, some authors have proposed strategies to mitigate anxiety during OSPE evaluation (23).

While some authors reported no significant differences between OSPE and traditional assessments (24), other studies demonstrated the benefits of an OSPE in Health Sciences (5, 14). In some way, it could be normal to obtain different results when employing different methodologies. And, until now, authors have not been able to confirm a correlation between OSPE and traditional evaluation (25). Nevertheless, a global survey including all the schools with implemented OSPE or OSCE reported that 80% of them stated that this methodology enhanced the achievement of teaching goals (1). According to the results of the OSPE, almost 94% of the students passed the exam on their first attempt with a good mean score (7.1/10). This reinforces the fact that OSPE enhances the assessment of psychomotor skills in healthcare careers (14). The “Clinical Examination” station had the poorest scores. The main difficulty of this station lies in the extensive knowledge that students need to acquire to pass it, including specific handling techniques, important anatomical details, and normal frequencies and body temperatures across four different species (dog, cat, cow, and horse). This finding is worrying, as one of the main objectives of the IVC subject is propaedeutics, which is the instruction aimed at gathering and interpreting the signs and symptoms of a patient to determine their health status and reach a diagnosis. Fal Dessai et al. (19), found similar results in “questionnaire stations”. Maybe the OSPE is not the best method to evaluate the contents relative to the “Clinical Examination” station, which belongs to the Knowledge domain rather than the Application one (15). Accordingly, “Surgery” and “Anesthesia”, focused only on dogs' and cats' psychomotor techniques, obtained the best scores.

It is important to recognize the limitations of the present study. Since this degree program is still being implemented at Universidad Europea de Madrid, and the IVC subject has been designed with the OSPE included, we cannot compare the results of students assessed through OSPE with those assessed through traditional methods. Although this comparison would have been of interest to corroborate the benefits of the OSPE, this has been previously reported in other healthcare grades (15, 25). Finally, there is no gold standard technique for evaluating skills in health sciences students. Ideally, the method should be valid, reliable, objective, and practical, allowing for differentiation between various types of students in a relaxed and standardized environment. Achieving an evaluation method that meets all these criteria is virtually impossible (25). However, the OSPE meets many of these criteria, and institutions where it has been implemented have reported significant benefits (14, 15). Therefore, the authors agree that this methodology should be expanded to other Veterinary Schools to enhance practical skills assessment strategies, despite the limited coordination among faculties and universities (5, 13). Such an expansion would also enable the evaluation of its long-term impact on the learning outcomes of students subjected to the OSPE.

## 5 Conclusions

The OSPE resulted in a more effective and valid assessment tool due to its ability to eliminate inter-examiner variation and bias. However, it is essential to ensure that the complexity of the various stations is comparable, thereby aligning the realism of the test with the subject's requirements and ensuring equitable evaluation across all stations. Furthermore, OSPE has been demonstrated to be a great tool for psychomotor skills evaluation. Nevertheless, the assessment of Knowledge content should be performed in combination with traditional examinations. In summary, OSPE implementation in Veterinary Medicine has demonstrated improvements in undergraduate skills and teaching and learning strategies. Therefore, OSPE should be more widely adopted in other universities.

## Data Availability

The original contributions presented in the study are included in the article/Supplementary material, further inquiries can be directed to the corresponding author.
