# Supplementary material for: Implementation of the first Objective Structured Practical Examination in veterinary medicine in Spain: a two-year experimental study
Source: Front Vet Sci. 2025 Aug 4;12:1618069. doi: 10.3389/fvets.2025.1618069 (PMC12359472; doi:10.3389/fvets.2025.1618069)

**Implementation of the first Objective Structured Practical Examination (OSPE) in Veterinary Medicine in Spain: a two-year experimental study**

Lara Carrasco^1^, Gema González^1^, Maria José Utrilla^1^, Laura Rico^1^, Antonio Magro^1^, Natividad Pérez^1^, Bárbara Martín-Maldonado^1^

^1^ Department of Veterinary, Biomedical and Life Sciences School, European University of Madrid, Villaviciosa de Odón, SPAIN

Corresponding author: Bárbara Martín-Maldonado (B.M.M., [barbara.martin-maldonado@universidadeuropea.es](mailto:barbara.martin-maldonado@universidadeuropea.es)). Biomedical and Life Sciences School, European University of Madrid, Calle Tajo s/n, Villaviciosa de Odón, 28670, Madrid, SPAIN.

**Supplementary Material 1. OSPE SATISFACTION SURVEY**

The objective of this survey is to collect data on the efficacy of the OSPE evaluation test for the Introduction to Clinical Practice course within the Degree in Veterinary Medicine at the European University of Madrid.

Your collaboration is essential to enhance the quality of our teaching and assessment in future editions. We therefore request your utmost sincerity and objectivity in your responses. To ensure an **open and honest feedback process**, we ask that you provide your **opinions anonymously**.

Please indicate your opinion on each of the items specified below:

1. The overall organization of the evaluation test was:

Poor Bad Neutral Positive Excellent

1. The organization of the Blood Sample Extraction station was:


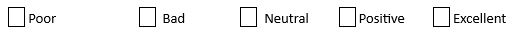


1. The organization of the Clinical Examination station was:


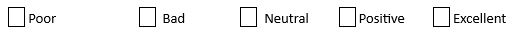


1. The organization of the Surgery station was:


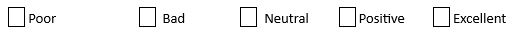


1. The organization of the Anesthesia station was:


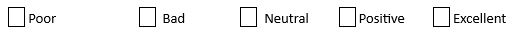


1. The information received before the OSPE was found to be:

Inadequate Adequate

1. The facilities where the OSPE took place were:

Bad Neutral Positive Excellent

1. The simulators used to carry out the OSPE were:


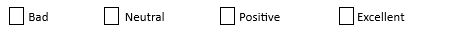


1. Please indicate whether you believe the stations accurately reflected actual practice:

Yes No

1. If not, indicate which station was not representative:

Clinical examination Blood sample extraction Anesthesia Surgery

1. The simulation activities carried out during the semester have effectively developed the competencies and knowledge necessary for the resolution of the OSPE:

Strongly Disagree Disagree Neither Agree nor Disagree Agree Strongly Agree

1. The use of simulators during the practical activities has helped you to acquire the competencies required for the practices in the shelter and farm:


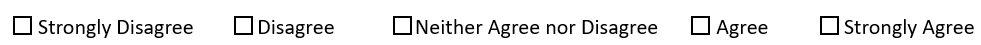


1. The simulators used in the practical activities seem to be sufficiently realistic for the acquisition of competencies:


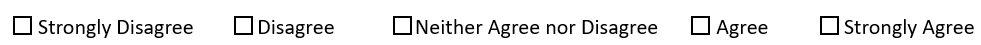


1. Please indicate which simulator has been the most beneficial in developing practical skills for use in a veterinary practice. Select one or two simulators.

Canine forelegs for blood sample extraction Ear pad for blood sample extraction

Canine heads for intubation Skin pads

Anesthesia machine Legs for stitching

Legs for bandaging Manikin

1. Please indicate which simulator has not assisted you in developing practical skills for use in a veterinary practice. Select one or two simulators.


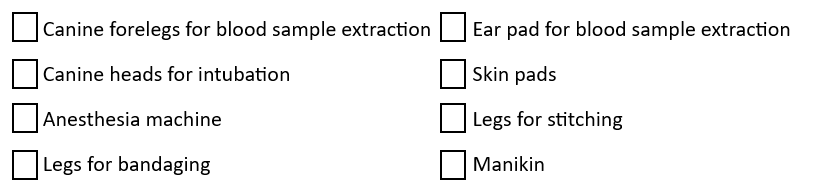


1. The clinical activities carried out in the shelter proved effective in helping you to pass the OSPE:


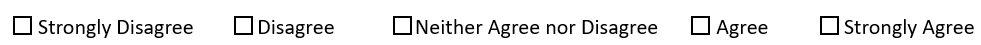


1. The duration of the internship at the shelter was:

Inadequate Insufficient Sufficient Adequate Excessive

1. The clinical activities carried out in the farm proved effective in helping you to pass the OSPE:


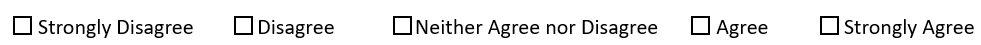


1. The duration of the internship on the farm was:


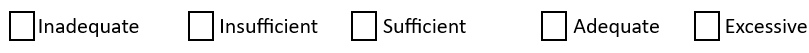


1. The OSPE has provided you with information on the skills required for your professional development:


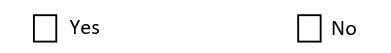


1. The time allowed to complete each OSPE station was:


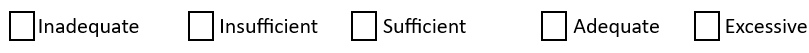


1. The duration of the OSPE and the number of stations were identified as follows:


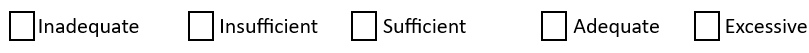


1. The OSPE is regarded as an effective and valuable tool for evaluating the practical skills acquired in this subject area:


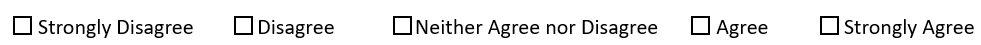


1. Please indicate whether you believe the system of assessment of practical skills for this subject is:

Inadequate Insufficient Sufficient Adequate

1. Please indicate your overall satisfaction with the OSPE:


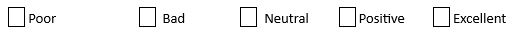


1. Please indicate the emotions you experienced during the OSPE. You may select up to three different emotions.


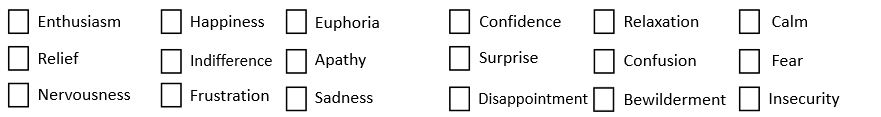


1. Please describe the emotions you felt after the test was completed. You may select up to three different emotions.


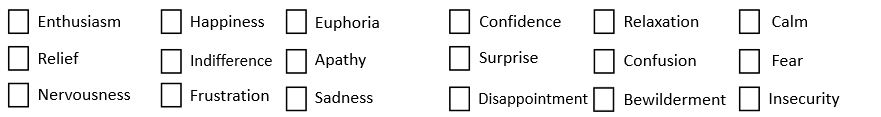

Supplement: Supplementary file 1 [file Table_1.docx]
